# 

**Published:** 1870-10

**Authors:** 


*CONTENTS:*

ORIGINAL COMMUNICATIONS:

ART. I. The Cell Theories of Huxley and Virchow. By I. N. Danforth, M.D. .......... 577

ART. II. Non-Ovarian Menstruation; with Cases and Some Remarks upon the Ovulation Theory. By A. Reeves Jackson, M.D. ...... 583

ART. III. Cases from the Surgical Clinic of Prof. Moses Gunn, of Rush Medical College. Reported by H. F. Chesbrough, M.D. ....... 594

ART. IV. Scorbutic Dysentery. By J. E. Thornburgh, M.D. ........... 598

ART. V. Case of Fracture of the Spine. By Daniel T. Nelson, Prof. Physiology, etc., Chicago Medical College .......... 600

ART. VI. Teaching the Deaf and Dumb to Speak. By David Greenberger, Ph.D. ........ 607

ART. VII. Cerebro-Spinal Meningitis. By J. E. Harvey, M.D. ..... 610

EPISTOLARY .......... 611

EDITOR'S BOOK TABLE .......... 618

Medical Diagnosis, with Special Reference to Practical Medicine. The Physical Exploration of the Rectum. The American Dispensatory.

EDITORIAL .......... 621

A word about Communications. News and Gossip. Notice of "Answer to Dr. Ruppaner." Obituary.

LOOT .......... 629

